# Optical coherence tomography evaluation of deep dentin crack removal techniques

**DOI:** 10.1016/j.jfscie.2022.100012

**Published:** 2022-08-12

**Authors:** Daniel Hovander, Grant Chyz, Yasushi Shimada, Junji Tagami, Alireza Sadr

**Affiliations:** a Biomimetics Biomaterials Biophotonics Biomechanics & Technology (B4T) Laboratory, Department of Restorative Dentistry, University of Washington, Seattle, WA; b Private Practice, Seattle, WA; c Cariology and Operative Dentistry Department, Graduate School of Medical and Dental Sciences, Tokyo Medical and Dental University, Tokyo, Japan

**Keywords:** Crack propagation, optical coherence tomography, particle abrasion, dentin, cracked tooth syndrome, diamond bur, carbide bur

## Abstract

**Background:**

This in vitro study used optical coherence tomography for noninvasive evaluation of the effectiveness of current clinical techniques to remove deep coronal dentin cracks.

**Methods:**

Standard dentin cracks were induced on the pulpal floor of 40 decoronated, extracted, sound human posterior teeth using a diamond disk, resembling cracks extending from marginal ridges. The specimens were randomly assigned to 1 of 5 treatment groups for crack removal using airborne-particle abrasion or a bur (fissure, small round, medium round, or tapered fine diamond). Optical coherence tomographic scans were obtained before and after the crack removal. Three-dimensional image registration analyzed the amount of dentin removed and the dimensions of cracks initiated or propagated in each treatment group.

**Results:**

Particle abrasion resulted in the smallest crack propagation in each dimension, which was significantly different from all bur groups (Mann-Whitney, *P* < .01). Removed dentin depth and width differed among burs (*P* < .05). All burs resulted in a degree of crack formation, and there was no difference in crack dimensions among bur groups (*P* > .05). The amount of removed dentin during crack treatment was the largest in the particle abrasion group, which was significantly different from those of the bur groups (Mann-Whitney, *P* < .005).

**Conclusions:**

Dental burs used with the purpose of removing pulpal floor dentin cracks induced new small cracks and extended existing cracks, and the volume of removed dentin depended on the shape and size of the bur. Air particle abrasion induced the fewest new cracks despite removing larger dentin volume.

## Graphical abstract

**Figure:**
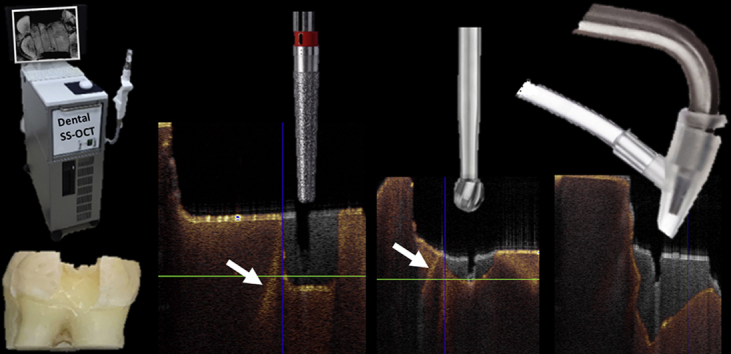

**Why Is This Important?**

Human teeth, particularly those with an old restoration or under high mechanical stress, may crack under cyclic functional loads over time. These cracks may extend longitudinally into dentin and over the pulp chamber. If not removed, dentin cracks can extend over time, leading to pulpal involvement and possible tooth loss. Practitioners may attempt to remove these cracks using dental burs. In our experiment, each bur type used to remove a large crack induced new smaller cracks, but the volume of removed dentin was significantly different between groups depending on the shape and size of the bur. Air particle abrasion was the only method that induced the least amount of new cracks despite removing a larger volume of dentin. Clinicians must be aware of crack propagation caused by various techniques and use clinical judgment to select an appropriate treatment method to prevent further propagation.

## Introduction

Mastication and parafunction create stresses and strains that act on the tooth structure. Intracoronal dental restorations variably weaken tooth structure, due to removal of cross bracing, creation of high-stress sharp line angles, retentive grooves or notches, irregular and sharp inconsistent surfaces induced with a dental drill, presence of nonbonded restorative materials, and stress induced by the shrinkage of bonded composite.^1^, ^2^, ^3^ In addition, when removing old restorations, the transmitted vibration may generate cracks or propagate existing cracks. These stresses may impose microcracks in the coronal tooth structure and can propagate, possibly resulting in tooth fracture.^3^, ^4^, ^5^, ^6^

Removal of tooth structure to facilitate placement of restoration often undermines tooth structure, increasing the risk of coronal cracking. These coronal cracks usually initiate at undermined proximal marginal ridges and propagate toward the center of the tooth in posterior teeth.^7^ Most such cracks may go undiagnosed until restoration replacement or until the cracks turn into a complete separation (fracture) or until pulpal-periodontal symptoms arise.^6^^,^^7^ In many cases, teeth with coronal cracks under existing restorations are asymptomatic, and by the time symptoms appear, the cracks develop significantly.^3^ When diagnosed properly and in a timely manner, depending on the nature and extent of the crack, a tooth with coronal crack may be saved. The restoration of such a cracked tooth can be costly, often involving root canal treatment followed by an indirect restoration such as a full-coverage crown, illustrating the large expenses that people with tooth fractures incur.^2^^,^^7^, ^8^, ^9^, ^10^ Many clinicians do not diagnose cracks in the enamel or dentin until the onset of physiological symptoms (cracked tooth syndrome).^2^^,^^11^ Cracks extending into the dentin are of particular importance owing to their role in catastrophic tooth fracture, propagation of periodontal lesions, bacterial leakage, and pulpitis.^2^^,^^3^^,^^11^ With the variability in formation, size, and location of dentin cracks together with the lack of a reference-standard diagnostic method, both diagnosis and treatment of this condition remain a challenge in clinical dentistry.^2^

A full-coverage crown has been a common treatment option for a cracked tooth, but cracks left under the crown may propagate in the future, resulting in endodontic complications or root fracture. A less invasive treatment approach for dentin cracks in a vital tooth includes the removal of the crack by a bur, followed by the placement of a bonded, often overlay, restoration with or without a base material or fiber-reinforced composite to limit further crack growth in function.^9^^,^^12^ However, 1 concern, particularly for a vital tooth, is the possibility of a crack opening up and further propagating under the stress, vibration, and heat generated by mechanical removal using a bur.^13^^,^^14^

Other methods of dentin crack removal with minimal or no vibration have gained attention. Airborne-particle abrasion with aluminum oxide is a mechanical pretreatment technique; it is a cost-effective method for surface roughening and can provide ultrafine mechanical tissue removal.^15^^,^^16^ Air abrasion is most commonly used to generate roughness in ceramic or composite restorations and increase the bond surface area, which might improve the bond strength of the surfaces.^15^, ^16^, ^17^, ^18^ Intraoral handpiece probes that provide precise aiming of the airborne particles have been introduced in the last 2 decades.^19^^,^^20^ Newer applications for this procedure include dentin caries removal and small cavity preparation.^19^^,^^20^ No study to date has looked at the potential application of the airborne-particle abrasion method for precise treatment of dentin cracks.

As of today, most dentin cracks are evaluated microscopically, with loupes, or unaided visualization.^21^ One challenge for the study of dentin cracks has been the detection and monitoring of these cracks. Diagnostic methods such as radiographs, methylene blue dye, and transillumination have been used for enamel and dentin cracks; however, limitations such as radiographs not providing adequate data on superficial cracks and the subjective nature of transillumination have been mentioned in previous studies.^21^^,^^22^ Even in laboratory studies, the microcracking of tooth structure as a result of mechanical removal by bur has been debated, since scanning electron microscopy (SEM) was not able to consistently document these cracks due to their small size and limitations of 2-dimensional microscopy, which requires physical cross-sectioning of the specimens.^23^ Researchers have measured dentin fatigue strength caused by various treatments as an indirect measure of possible flaws introduced as a result of mechanical removal of dentin.^13^^,^^14^

Less invasive methodologies for crack detection are being explored. Quantitative light-induced fluorescence has shown promise in the detection of both caries and dental cracks. This nonradiation fluorescence allows depth assessment, a parameter not measured by other assays such as methylene blue dye and transillumination.^24^ Similarly, OCT has gained popularity for noninvasive cross-sectional studies of caries, cracks, and restorations.^25^, ^26^, ^27^, ^28^ OCT can be used to characterize the dentin crack in real time. Sophisticated image analysis can create 3-dimensional (3D) quantitative measurement of the cracks with excellent specificity and sensitivity.^29^^,^^30^ This study aims to use OCT as an objective diagnostic tool for dentin cracks. There are limited data and no widely accepted clinical protocol for treatment of dentin cracks. This is the first study to our knowledge to use advanced OCT technology for direct assessment of dentin microcracking. The null hypothesis was that there was no difference in the removal of dentin crack among the treatments with 4 types of dental burs and airborne-particle abrasion.

## Methods

**Figure 1:**
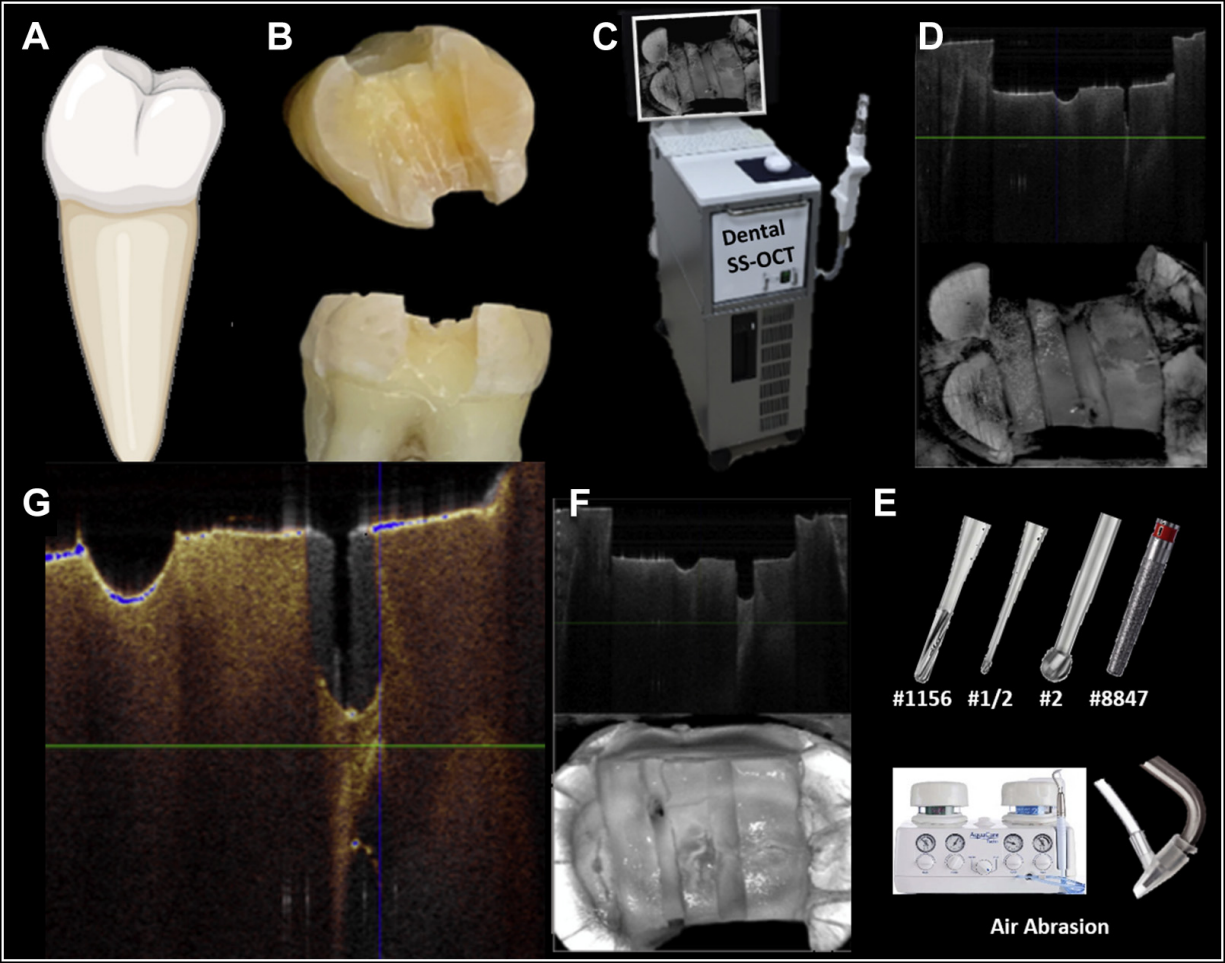
Methodology of experiment. **A.** Forty posterior teeth randomly assigned to 1 of 5 treatment groups. **B.** Specimens were decoronated with diamond saw and occlusal and peripheral enamel was removed with a wheel-shaped diamond bur and electric handpiece at 150,000 rpm with water. A diamond disk with a cutting head of 140 μm was used to simulate a coronal crack approximately 0.7 mm in depth. A flame bur was used to create a mesiodistal guide notch. **C.** The specimens were scanned using Dental SS-OCT (Yoshida). **D.** Cross-sectional and occlusal pretreatment optical coherence tomography (OCT) were collected. **E.** Each specimen underwent 1 of the 5 treatments to remove the initial dentin crack. **F.** Posttreatment OCT scans were collected to evaluate the initial crack treatment. **G.** Amira 3-dimensional software (Version 6.7; ThermoFisher Scientific) was used to superimpose OCT scans and analyze the dimensions of crack propagation or new crack formation for each treatment.

An overview of the experimental design and imaging processes in this study are schematically presented in Figure 1.

### Specimen preparation

Forty extracted human posterior teeth were included and randomly assigned to 1 of the 5 treatment groups (n = 8). Sample size was determined through power analysis based on the pilot study of crack size among the groups. Criteria for teeth selection included classification of posterior teeth with no existing visible crack, caries, or restoration. Teeth were collected from several dentists and oral and maxillofacial surgeons across the Seattle, Washington, area. The teeth were properly stored in 100 mL of distilled water with the addition of thymol crystals to maintain the microhardness throughout the experiment duration.^31^ This study was conducted according to the ethical guidelines set by the University of Washington Institutional Review Board, which waived the approval requirement for the use of deidentified human teeth extracted as a part of routine dental treatment.

**Figure 2:**
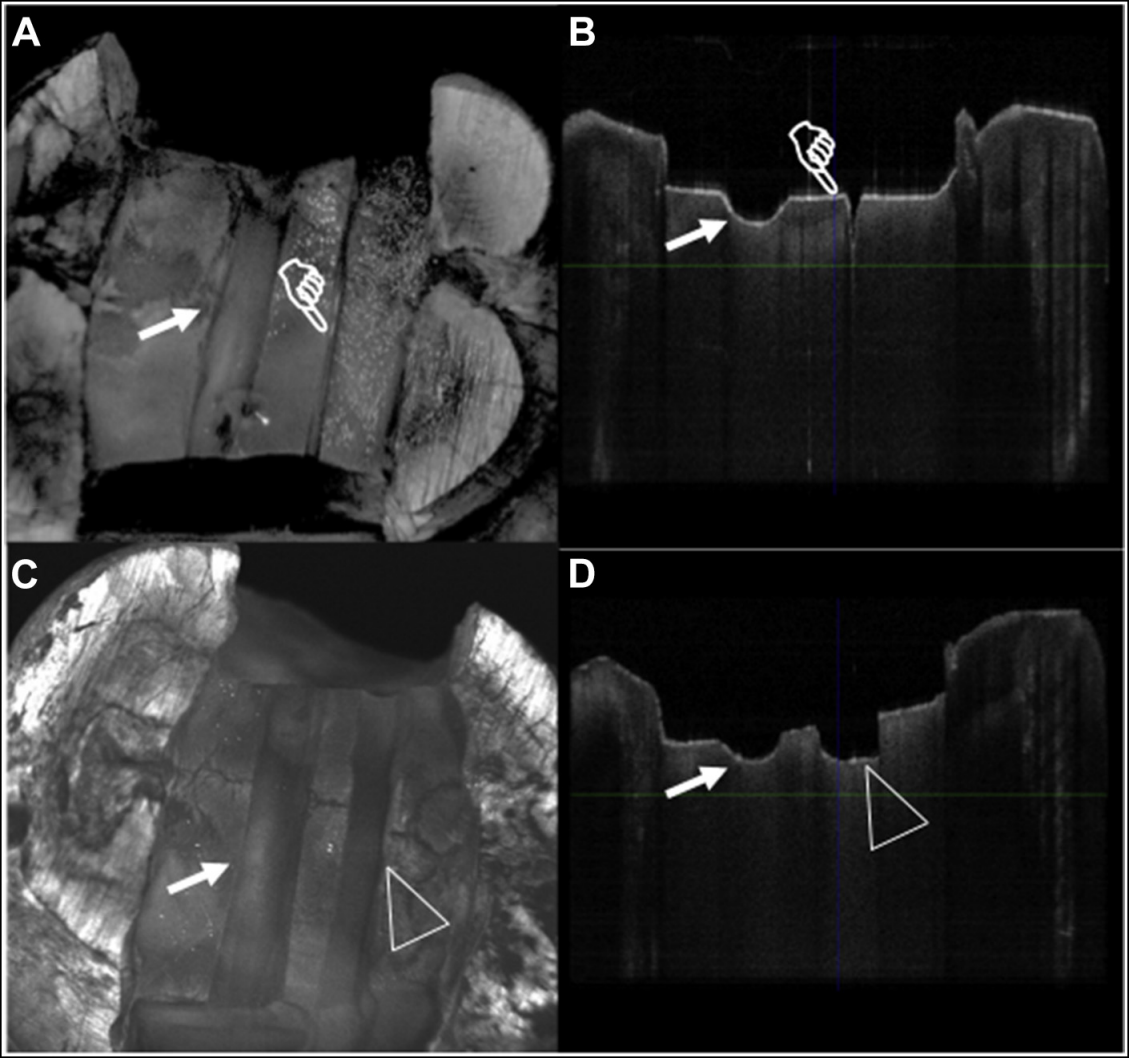
**A.** Occlusal pretreatment optical coherence tomographic (OCT) scan. Note the pretreatment crack was created by diamond disk (finger pointer) and guide notch (arrow pointer). **B.** Cross-sectional pretreatment OCT scan showing guide notch and pretreatment crack. **C.** Occlusal posttreatment OCT scan showing guide notch (solid arrow) and treatment site (triangle). **D.** Cross-sectional posttreatment OCT scan showing guide notch (solid arrow) and treatment site after crack removal with a no. 1156 bur (triangle).

At the time of the experiment, the cusps on all teeth were reduced by 1 mm, and a 4 mm wide and 2 mm deep channel was created at midcoronal dentin with 2 proximal boxes using a wheel-shaped diamond bur attached to an electric dental handpiece (Ti-Max Z95L; NSK) at 150,000 rpm under water spray cooling. A guide notch was then created over the pulpal floor dentin extending mesiodistally using the tip of a flame-shaped 30-μm grit fine diamond bur (no. 8862, Komet) as a reference landmark. An initial OCT scan was collected after the placement of the guide notch, which was denoted as the baseline. The OCT scanning procedure is explained in detail below. A diamond disk (Medium HyperFlex Double Sided Diamond Disk; Brasseler) with a cutting head thickness of 140 μm was then used to simulate a mesiodistal dentin crack with an approximate depth of 0.7 mm on the pulpal floor, about 1 mm away from the guide notch. A second OCT scan was collected and denoted as pretreatment OCT (Figure 2).

### Experimental groups

After baseline and pretreatment OCT scans, each experimental group underwent 1 of the 5 treatments aimed at removing or diminishing the baseline simulated crack. The treatment groups were

1. Fissure carbide bur (no. 1156; Komet) attached to a high-speed electric handpiece (CA 1:5 L Micro Series; Bien Air) at 150,000 rpm with water spray.
2. Round carbide bur no. ½ attached to the high-speed electric handpiece at 150,000 rpm with water spray.
3. Airborne-particle abrasion: 53-μm aluminum oxide powder, 0.6-mm diameter nozzle, and up to 5 bar pressure with water flow (Aquacare; Medivance Instruments).
4. Round carbide bur (no. 2; Komet) attached to the high-speed electric handpiece at 150,000 rpm with water spray.
5. Tapered round-end fine diamond bur (no. 8847; Komet) attached to the high-speed electric handpiece at 150,000 rpm with water spray.

All treatments were performed by the same calibrated operator (D.H.), who intended to remove the crack completely under simulated clinical conditions with ×3.5 dental loupe magnification and standard illumination. The specimens were kept hydrated at 37 °C in an incubator between the experimental steps. After the treatment in each group was completed, a final OCT scan was obtained and was denoted posttreatment OCT (Figure 2 C, D).

### OCT scanning and image analysis process

3D OCT scans were obtained from the occlusal surface of the specimens over a 10- × 10-mm window by using a prototype swept-source OCT system (Yoshida Dental) with the center and range wavelengths of 1,310 nm and 140 nm, respectively, and a frequency of 50 kHz. The system optical resolution was 11 μm in depth, for a medium with a hypothetical refractive index of n = 1. The OCT handheld scanning probe was placed at a predetermined distance from the specimen, projecting the scanning beam to the experimental surface. A 3D scan with this configuration can be obtained in less than 4 s over an optical depth of 8 mm and 400 × 400 × 2048 voxel data.

The 3D data set was then imported into the analysis software (Amira Version 6.7). The pretreatment and posttreatment scans for each specimen were aligned and registered in 3D manually by visualizing the guide notch and then fine-tuned using the image registration function of the software to enable accurate comparisons between the scans at each spatial location (Figure 2).

The comparisons were performed on 5 standard regions of interest (ROIs) on each specimen, defined as the dentin area on cross-sectional slices 0.5 mm through 1 mm apart covering the mesiodistal length of the initial crack. In total, 7 variables were measured for each specimen from the superimposed OCT scans.

To ensure standardization of the initial simulated crack among the groups, the initial crack depth and initial crack width were measured using the software on the central cross-sectional ROI.

For comparison of the amount of dentin structure removed with each treatment method, the removed dentin depth and removed dentin width variables were measured through the subtracting comparison of the registered pretreatment and posttreatment scans at each ROI.

Dentin crack initiation and propagation index (DCIPI) was defined as the dimension of the longest new or propagated crack observed after removal of the initial (pretreatment) crack in the ROI in each orientation: *DCIPI*_*MD*_ (X, Mesiodistal), *DCIPI*_*BL*_ (Y, Buccolingual), and *DCIPI*_*Depth*_ (Z, axiopulpal). If no crack was observed in the ROI, a value of 0 was recorded.

### Statistical analysis

The frequency of posttreatment crack observation among groups was compared using the Pearson χ^2^ test. All measured variable data were subjected to normality tests using Kolmogorov-Smirnov *Z* to determine the appropriate statistical comparison method. Accordingly, 1-way analysis of variance was used to analyze the initial crack depth and initial crack width across groups.

Removed dentin depth, removed dentin width, DCIPI_MD_, DCIPI_BL_, and DCIPI_Depth_ were compared by Kruskal-Wallis tests among all groups, followed by the Mann-Whitney *U* test for pairwise comparisons with Bonferroni correction. All analyses were carried out using SPSS 16.0 software (SPSS) at a preadjustment significance level of α = 0.05.

## Results

**Figure 3:**
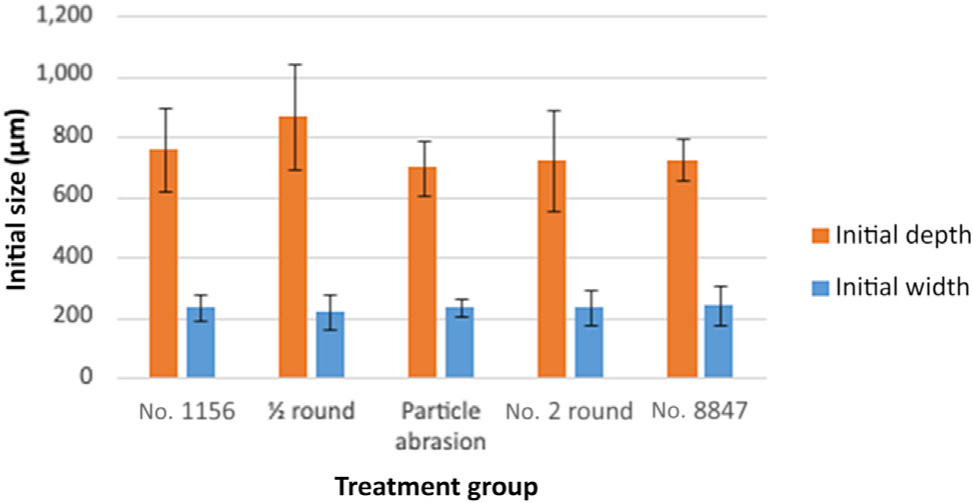
Bar chart comparing the initial size of crack in μm by treatment group on the category axis. There was no statistically significant difference among the groups for each variable (*P* > .05).

The initial crack depth and initial crack width across groups were 700 through 800 μm and 200 μm, respectively, and are presented in Figure 3. One-way analysis of variance showed that both variables were not significantly different among groups with *P* = .104 and *P* = .082, respectively.

**Figure 4:**
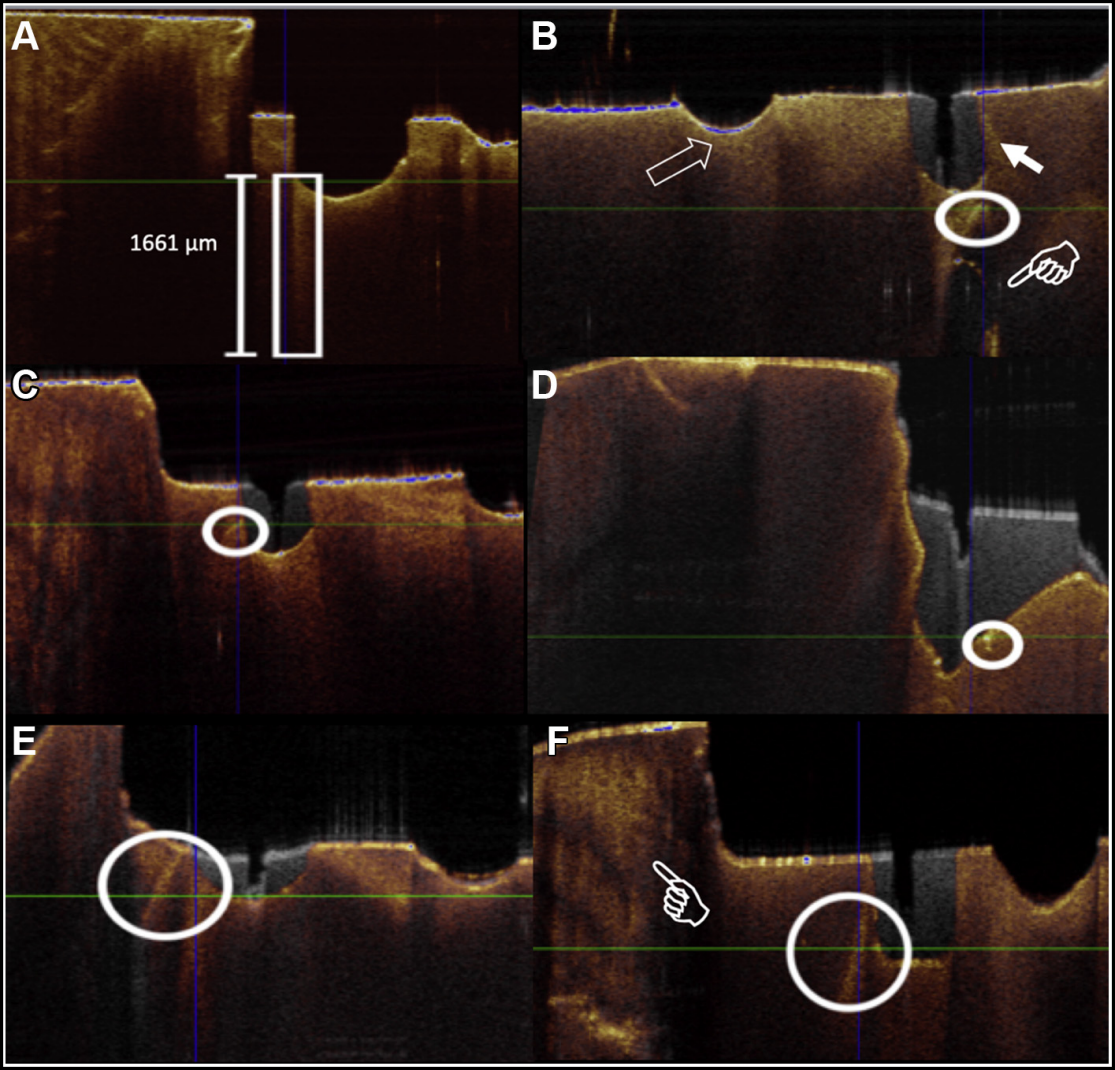
**A.** No. 1156 exhibiting one of the deepest dentin cracks (rectangle) induced across treatment groups. Crack dimensions (dentin crack initiation and propagation index [DCIPI]_Depth_ × DCIPI_Buccolingual_ × DCIPI_Mesiodistal_) are 1,661 μm × 38 μm × 74 μm. **B.** In no. 1156 treatment, new crack propagating from the base treatment site (solid arrow) measured 426 μm × 100 μm × 100 μm. Note the new extending crack is in proximity with the pulpal horn (finger pointer) away from the guide notch (arrow). **C.** ½ round carbide treatment showing a new crack (117 μm × 55 μm × 55 μm) extending laterally from treatment site. **D.** Particle abrasion treatment, with small crack extending occlusogingivally from treatment site with dimensions of 114 μm × 80 μm × 100 μm. Note the difference in depth and width removal compared with bur treatment groups. **E.** New crack created from treatment site of no. 2 round, starting occlusally and traveling interproximal. Crack dimensions were 1,198 μm × 69 μm × 73 μm. **F.** Dentinoenamel junction (finger). No. 8847 resulted in a new crack (1,228 μm × 99 μm × 460 μm) created from base of treatment site propagated toward the gingiva.

The representative OCT image results of all groups are presented in Figure 4. In each OCT panel, 2 images are included; the grayscale image represents the pretreatment scan, which included a guide notch in the center and the induced crack, and the colored super imposing scan represents the posttreatment scan. Overall, the frequency of the observed dentin cracks on the ROIs were 32 in the no. 1156, ½ round, and no. 8847 groups; 30 in no. 2 round; and 22 in the particle abrasion group. Pearson χ^2^ showed a significant difference in frequency of the posttreatment crack observation among groups with χ^2^_4_ (N 200) = 9.77, *P* < .05.

In the no. 1156 group (Figure 4A), the posttreatment scan illustrated complete removal of the original crack. However, a deep coronal crack was created during the removal process in this specimen. This new crack extended from the treatment site (left side of the guide notch) toward the gingival portion of the specimen. DCIPI_Depth_ of this crack was measured to be 1,661 μm. Figure 4B illustrates a more comprehensive analysis of the no. 1156 treatment group with the average DCIPI_Depth_ being 247 μm. Specifically, Figure 4B exhibited a crack propagating from the base of the treatment site toward the pupal horn with a DCIPI_Depth_ of 426 μm. The ½ round representative specimen (Figure 4C) displayed a posttreatment crack extending in the occlusogingival direction. The dimensions of the crack (DCIPI_Depth_ × DCIPI_BL_ × DCIPI_MD_) were approximately 426 μm × 100 μm × 100 μm.

Figure 4D presents the particle abrasion treatment, which resulted in a wide and deep area of dentin removal and fewer smaller posttreatment cracks. The no. 2 round treatment group, represented by Figure 4E, exhibited a generally wider but shallower area of dentin removal, but posttreatment cracks were still visible in this group. A posttreatment crack originated near the occlusal surface and propagated toward the buccal surface. Dimensions of crack were 1,198 μm ×119 μm ×189 μm. The no. 8847 treatment displayed a narrower, but deeper treatment site compared with the no. 2 round. Figure 4F exhibits a crack propagating in the occlusoginvigal direction with approximate dimensions of 1,228 μm × 99 μm × 460 μm, respectively. Table 1 is a comprehensive analysis of DCIPI including DCIPI_MD_, DCIPI_BL_, and DCIPI_Depth_. Overall, the largest cracks were in the axiopulpal depth dimension (DCIPI_Depth_), followed by the mesiodistal dimension (DCIPI_MD_). Kruskal-Wallis analysis showed a significant difference among groups in all crack variables (*P* < .05).

**Table 1:** Comprehensive analysis of treatment groups including removed depth, removed width, and DCIPI∗.

Variable	Groups	Mean (SD)	95% CI	Mean Rank†
Removed Depth	No. 1156	314.5 (285.6)	221.9 to 407.1	99.15^a^
	½ round	160.9 (183.5)	98.8 to 223	59.14^b^
	Particle abrasion	1,123.7 (338.5)	1,015.4 to 1,231.9	169.28^c^
	No. 2 round	164.2 (124.6)	122.0 to 206.3	69.11^b^
	No. 8847	180.9 (127.5)	140.1 to 221.7	77.02^b^
Removed Width	No. 1156	852.7 (157.1)	802.4 to 902.9	118.32^a^
	½ round	407.6 (81.7)	381.5 to 433.7	23.38^b^
	Particle abrasion	1,174.9 (288.0)	1,082.8 to 1,267.0	161.95^c^
	No. 2 round	991.1 (223.6)	919.6 to 1,062.6	138.70^d^
	No. 8847	561.4 (74.0)	537.7 to 585.1	60.15^e^
DCIPI_M__esiodistal_	No. 1156	94.2 (74.6)	70.3 to 118.0	104.70^a^
	½ round	88.5 (72.0)	65.1 to 111.8	100.10^a^
	Particle abrasion	53.1 (66.5)	31.8 to 74.4	70.30^b^
	No. 2 round	127.3 (117.6)	89.7 to 164.9	114.48^a^
	No. 8847	122.0 (119.3)	83.9 to 160.2	110.42^a^
DCIPI_B__uccolingual_	No. 1156	65.1 (51.2)	48.7 to 81.5	107.48^a^
	½ round	54.6 (35.5)	43.3 to 66.0	98.00^a^
	Particle abrasion	32.8 (34.0)	21.9 to 43.7	69.45^b^
	No. 2 round	67.8 (54.4)	50.4 to 85.2	112.35^a^
	No. 8847	64.6 (43.1)	50.7 to 78.6	112.33^a^
DCIPI_Depth_	No. 1156	247.4 (312.8)	147.4 to 347.4	110.20^a^
	½ round	186.4 (212.2)	118.5 to 254.2	99.88^a^
	Particle abrasion	86.8 (94.6)	56.5 to 117.0	66.98^b^
	No. 2 round	273.0 (326.5)	168.5 to 377.4	109.95^a^
	No. 8847	257.3 (276.5)	168.9 to 345.7	115.50^a^

∗ DCIPI: dentin crack initiation and propagation index.

† Mean Rank: Kruskal-Wallis mean rank. Different superscript letters in the column indicate statistically significant difference between the groups Mann-Whitney *U* pairwise comparisons (*P* < .05).

The dimensions of the removed dentin structure as a result of crack treatment were largest in the particle abrasion group as exhibited by the average depth removal of 1,124 μm and an average width removal of 1,017 μm (Table 1). The average ½ round treatment dimensions were 139 μm × 460 μm, which was the smallest volume of removed dentin across treatments. Both the ½ round and no. 2 round had a similar depth removal of approximately 139 μm (Table 1).

The mean DCIPI_MD_ ranged from 53 μm to 127 μm across treatment groups. The largest average DCIPI_MD_ was 127.3 μm in the no. 2 round treatment. In contrast, particle abrasion exhibited the smallest average DCIPI_MD_ of 53.1 μm, which was significant compared with all bur groups with *P* <.005. No other DCIPI_MD_ comparisons were significant (*P* > .05).

The no. 2 round resulted in the largest mean DCIPI_BL_ of 68 μm. In contrast, the smallest mean DCIPI_BL_ was 33 μm shown by the particle abrasion treatment. The average DCIPI_BL_ produced by particle abrasion was nearly one-half that of the bur treatments, excluding ½ round. The DCIPI_BL_ in the particle abrasion group was statistically significantly different from all other groups with *P* < .005.

The greatest range of DCIPI_Depth_ was displayed in the no. 1156 treatment, with crack depth varying from 147 μm through 374 μm. In comparison, particle abrasion that exhibited the narrowest range from 57 μm through 117 μm. However, across all treatment groups, mean DCIPI_Depth_ ranged from 87 μm through 273 μm. The largest average DCIPI_Depth_ (273 μm) was produced by the no. 2 round bur, whereas the smallest average DCIPI_Depth_ (87 μm) occurred in the particle abrasion group. DCIPI_Depth_ in each bur treatment group was significantly different from the particle abrasion (*P* < .005).

A summary of all statistical analyses is presented in Table 2. In addition, each variable data are presented in the form of box plots in the Appendix A figures.

**Table 2:** Statistical analysis of *P* values from the pair comparisons using the Mann-Whitney *U* test.

Variable	Groups	No. 1156	½ Round	Particle Abrasion	No. 2 Round
Removed Depth	½ round	0.000∗	NA†	NA	NA
	Particle abrasion	0.000∗	0.000∗	NA	NA
	No. 2 round	0.002∗	0.233	0.000∗	NA
	No. 8847	0.048∗	0.074	0.000∗	0.74
Removed Width	½ round	0.000∗	NA	NA	NA
	Particle abrasion	0.000∗	0.000∗	NA	NA
	No. #2 round	0.011∗	0.000∗	0.002∗	NA
	No. 8847	0.000∗	0.000∗	0.000∗	0.000∗
DCIPI_Mesiodistal_‡	½ round	0.609	NA	NA	NA
	Particle abrasion	0.004∗	0.010∗	NA	NA
	No. 2 round	0.324	0.214	0.002∗	NA
	No. 8847	0.588	0.460	0.002∗	0.460
DCIPI_Buccolingual_	½ round	0.475	NA	NA	NA
	Particle abrasion	0.003∗	0.010∗	NA	NA
	No. 2 round	0.684	0.246	0.002∗	NA
	No. 8847	0.708	0.233	0.001∗	0.233
DCIPI_Depth_	½ round	0.369	NA	NA	NA
	Particle abrasion	0.001∗	0.004∗	NA	NA
	No. 2 round	0.961	0.405	0.002∗	NA
	No. 8847	0.595	0.179	0.000∗	0.179

∗ indicates statistical significance with *P* < .05.

† NA: not applicable.

‡ DCIPI: dentin crack initiation and propagation index.

## Discussion

Microcracks within the coronal tooth structure may propagate, possibly resulting in tooth fracture.^3^^,^^4^^,^^11^ These fractures may result in painful and costly procedures including extraction, root canal therapy, and crowns.^7^^,^^9^ The recommended method for removal of dentin crack is drilling it out; however, crack propagation has been a noted concern because of the heat generated and mechanical stress imposed on the tooth structure.^13^^,^^14^

We used OCT for the first time for comprehensive analysis of dentin microcracks. OCT is a noninvasive cross-sectional imaging technique that detects backscattered light along with echo time delay.^2^ OCT can detect cracks due to the contrast in light scattering caused by dentin and enamel cracks. This method has been validated by several studies.^2^^,^^25^^,^^29^ Although our OCT study was conducted in vitro, evidence for its intraoral application is growing.^32^ Other diagnostic assays, such as microcomputed tomography, are not appropriate alternatives due to the lack of resolution for fine cracks.^21^ Similarly, SEM requires slicing of specimen and eliminates baseline comparisons. In fact, the formation of microcracks as a result of dentin mechanical removal has been a theoretical notion based on the observation of dentin fatigue strength reduction after treatment rather than direct observations since SEM has been inconsistent in producing measurable results.^13^^,^^14^^,^^23^

One limitation of our study was the creation of a simulated crack instead of a naturally occurring crack under fatigue loading; however, creating standard dentin natural cracks to compare across groups would be challenging. The results presented in Figure 3 suggested that the simulated cracks were comparable at the baseline, enabling comparison among crack removal methods. Simulated cracks have more dentin loss and are possibly blunter at the crack tip than natural cracks. It was speculated that natural cracks would propagate even further under vibration owing to a sharper crack tip.

Electric handpieces were primarily used by the operator throughout the duration of the experiment. Less crack propagation was expected compared with conventional air-driven turbines because of less vibration at higher torques. The literature has also reported on the effect of bur selection on dentin surface roughness. In general, finer grits are expected to create a smoother surface.^13^^,^^14^ In our study, standard steel burs, carbide burs, and fine-grit diamonds were included. Although there were differences in the amount of dentin removal with each bur that could be attributed to the geometry of the burs, these treatment groups were not statistically significant when analyzing dentin crack initiation and propagation or DCIPI. It was reported that the direction of cutting in relation to the orientation of dentin was an important factor in the possible propagation of the cracks.^13^ This is because dentin is an anisotropic substrate with varying dentinal tubule orientation and collagen-mineral content.^14^ It appears that cracks propagate regardless of the bur type at the pulpal floor dentin where a major dentin crack had previously developed under a wedging force. This observation is also in line with the literature that a reduction in the dentin resistance to fatigue crack growth was observed in deeper dentin, as deeper dentin is more brittle.^13^^,^^14^^,^^33^ The initial preparation of sound dentin did not create as many cracks as was caused by attempts to remove the simulated cracks, based on OCT imaging. This observation indicates the particular challenge associated with treating a preexisting crack in deep dentin.

The particle abrasion treatment resulted in significantly smaller cracks across every dimension (DCIPI_Depth_ × DCIPI_BL_ × DCIPI_MD_) compared with bur treatments, suggesting that particle abrasion may not induce the same mechanical stress as rotary instruments. This result rejected our null hypothesis of no difference in crack removal across treatment groups. In addition, the frequency of cracks and treatment dimensions were different among the groups. The technique in our study is a new implementation of particle air abrasion with improved control due to smaller nozzle size as well as an adjustable simultaneous water spray. Although the crack frequency and size was diminished by with particle abrasion, the dimensions of removed dentin were much greater than with bur treatments, which indicates less control while removing the initial dentin crack compared with bur treatments. The studies on the dentin fatigue strength indicated that both bur and air abrasion treatments resulted in reduced fatigue strength of dentin, which is in line with the observation of crack formation in all groups.^13^^,^^14^

Our study has limitations. Dentin properties in extracted teeth could depend on various factors and may not necessarily represent the behavior of dentin in a vital tooth or under different clinical situations. Thin grooves were used to simulate cracks, and true cracks were not used. We were unable to create true standardized cracks, ultimately settling on the use of a thin diamond disk, with the assumption that the disk would leave a comparatively blunt crack that would lessen the effect of bur and handpiece vibration rather than amplify it. The investigations of an appropriate treatment method for dentin cracks should continue. The use of carbon dioxide lasers in the dental field has been a growing concept in dental hard- and soft-tissue treatment.^34^^,^^35^ Our research will continue to examine crack formation in dentin after various potential crack treatments such as laser treatment and application of bioactive materials. The prototype OCT system used in our study is capable of intraoral imaging; therefore, clinical analysis of these dentin cracks is a possibility. OCT along with sophisticated image analysis is opening a new horizon in research on the formation, propagation, and treatment of cracks in dentin.

## Conclusions

Each bur type used throughout this experiment induced new cracks, but the volume of removed dentin was significantly different among the groups depending on the shape of the bur, a variable that should be considered for clinical situations. Air particle abrasion induced the least amount of new cracks despite removing larger dentin volume and should be given consideration when applicable. Clinicians must be aware of crack propagation caused by various techniques and use clinical judgment to select an appropriate treatment method to prevent further propagation.
