# Supplementary material for: Optical coherence tomography evaluation of deep dentin crack removal techniques
Source: JADA Found Sci. 2022 Aug 12;1:100012. doi: 10.1016/j.jfscie.2022.100012 (PMC13229053; doi:10.1016/j.jfscie.2022.100012)

**Appendix**

Box plots representing the distribution of all crack data in all groups A: CIPI_Depth_ ;B: DCIPI_BL_;C: DCIPI_MD_

**A**


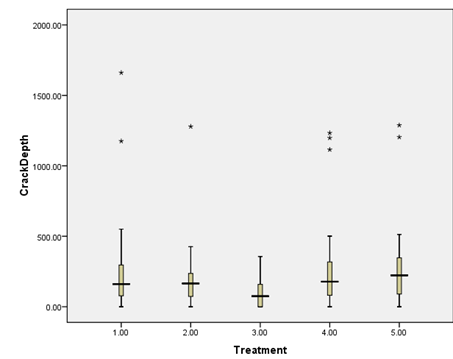


**B.**

**
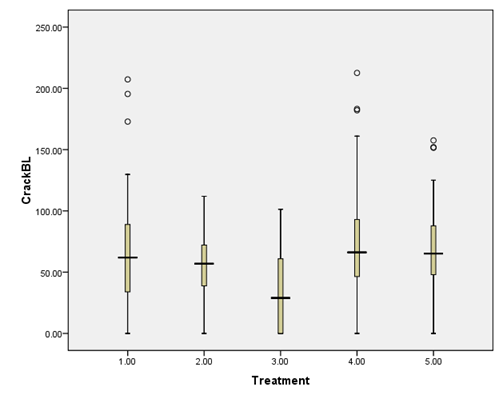
**

**C.**


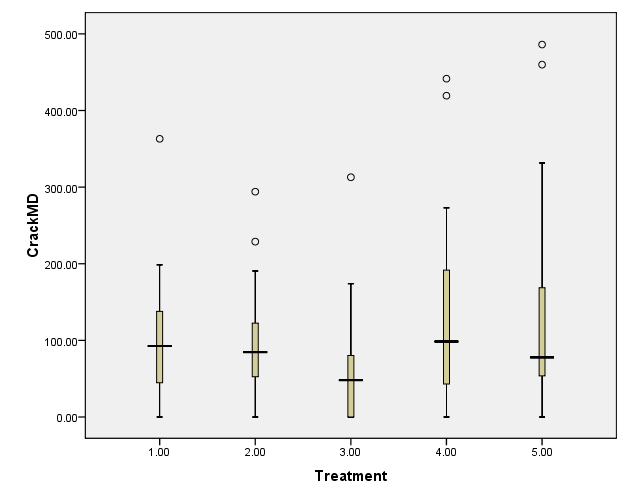

Supplement: Appendix [file mmc1.docx]
